# A New Integrated Knowledge Management & Data Analysis Platform for Cell Line Development & Cell Culture R&D

**DOI:** 10.1186/1753-6561-9-S9-P63

**Published:** 2015-12-14

**Authors:** Jens Niklas, Julia Retey, Steffen Fehrmann, Ludwig Macko, Thomas Hartsch, Ben Adamczyk

**Affiliations:** 1 Genedata AG, Basel, 4053,Switzerland; 2 Genedata Inc, Lexington, MA, 02421, USA

## Background

New technologies such as next generation sequencing (NGS) and -omics have changed the way in which cell line development, cell line engineering and cell culture R&D are generally performed. However, genomic and molecular data are often scattered in different locations, in different formats, and not easily accessible. Additionally, data quality issues may be inadequately tracked, leading to suboptimal decisions and increasing the risk of project failure. Complex workflows may require different tools or may not be supported by available software solutions.

In order to reduce the time and costs associated with cell line development and cell culture R&D projects, intelligent bioinformatics tools and knowledge management systems are required ensuring collaboration and knowledge sharing among research groups and sites.

## Results

**Figure 1:**
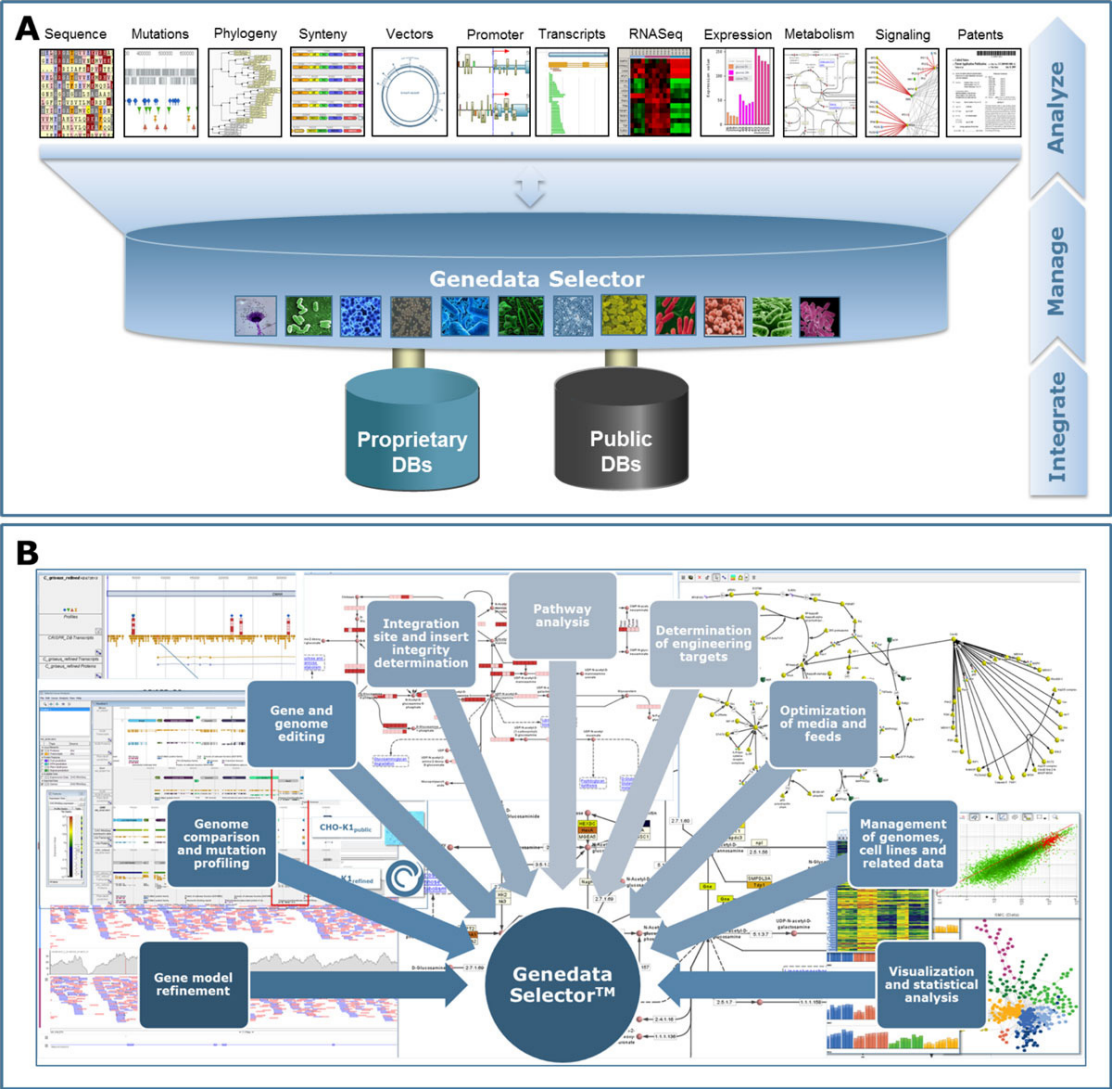
**A**. Scheme of the open and scalable platform, providing a comprehensive view from nucleotides via pathways to patents. B. Excerpt of applications for cell line development and cell culture R&D supported by the platform.

To address these challenges, we developed a single, integrated knowledge management and analysis platform, Genedata Selector™ (Figure 1A). The enterprise platform integrates public and proprietary genome and "omics" data from different sources and is highly scalable, being able to handle hundreds of genomes and related information. Additionally, all publicly available CHO cell lines and the Chinese hamster genome with refined gene models, improved functional annotation, transcription factor binding site and pathway information are integrated in this one platform.

Due to this open, integrated and versatile platform for all NGS and omics technologies, many applications are supported. This supports streamlining of R&D processes and makes valuable information (such as genomes, cell lines and related experimental data) readily accessible to different teams, thereby avoiding duplication of research efforts (Figure 1B). To guarantee that the information in the system is always up to date, numerous different databases can be automatically scanned, and the information updated in the system.

Standardized workflows in the system enable, e.g. the processing and analysis of RNASeq data, to gene prediction and gene model refinement, and generation of fully annotated genomes from raw resequencing data. Users have access to the best gene models for proprietary cell lines, building the foundation for successful genome-based R&D. Differences between cell lines on the genome, protein, pathway level, etc. can be elucidated easily through interactive analysis tools, enabling the understanding of the underlying biology through a single, integrated view.

For cell line engineering, sophisticated signaling pathway analysis tools were integrated in the platform to enable efficient prioritization of engineering targets for improving protein production (e.g. focusing on apoptosis or stress control, protein secretion, glycosylation or genome stability). When targets have been identified, the next step is to define the gene editing strategy. Genedata Selector supports all gene editing technologies. For instance, CRISPR/Cas9 target sequences can be calculated in any genome (de novo or resequencing data), and target gene sequences and uniqueness of editing sites can be interactively analyzed. Errors during target sequence design are reduced and the success rate of gene/genome editing projects can be increased significantly.

Another high-value application, which is now possible with new NGS technologies, is the accurate determination of integration sites and the integrity of inserts in clones. Here, we have applied the platform to localize insertions in proprietary gene models through automated bioinformatics pipelines for processing and analysis of sequencing data. This avoids costly propagation of clones with wrong/disrupted inserts.

Rational optimization of cell lines, media and process parameters is another issue which we have addressed. Integrated genomics/omics data analysis on pathways are provided in the platform, enabling users to identify novel relationships in experimental data and to understand molecular differences between cell lines, cultivation conditions and time points. In particular, the integrated view provided through the platform allows the rapid interpretation of complex datasets. This provides strong support for rational optimization of media and feeds by facilitating discovery of limitation or accumulation of metabolites and other key factors which have to be optimized to avoid costly trial and error.

All these applications are supported by an integrated rich statistical toolbox allowing the easy identification of biological functions and pathways which show significant differences between cell lines/clones/process conditions.

## Conclusions

We have developed an integrated software platform, Genedata Selector, to streamline cell line engineering, cell line development and cell culture optimization R&D in biopharmaceutical production processes. The platform helps to reduce the high costs and long development times currently required for these processes. Designed as an enterprise system, the platform ensures knowledge sharing and collaboration among research groups and sites, avoiding unnecessary duplication of research efforts. The development of highly competitive proprietary cell lines and processes is supported, increasing the success rate of R&D projects

